# Co-application of ACC deaminase-producing rhizobial bacteria and melatonin improves salt tolerance in common bean (*Phaseolus vulgaris* L.) through ion homeostasis

**DOI:** 10.1038/s41598-022-26084-3

**Published:** 2022-12-21

**Authors:** Mozhgan Alinia, Seyed A. Kazemeini, Ali Dadkhodaie, Mozhgan Sepehri, Vahid A. Jahandideh Mahjenabadi, Syeda F. Amjad, Peter Poczai, Doaa El-Ghareeb, Mohamed A. Bassouny, Ahmed A. Abdelhafez

**Affiliations:** 1 Plant Production and Genetics Department, Shiraz University, Shiraz, Iran; 2 Soil Science Department, Shiraz University, Shiraz, Iran; 3 Agriculture Research, Education and Extension Organization, Soil and Water Research Institute, Karaj, Iran; 4 Department of Botany, University of Agriculture Faisalabad, Faisalabad, Pakistan; 5 Botany Unit, Finnish Museum of Natural History, University of Helsinki, 00014 Helsinki, Finland; 6 Agriculture Genetic Engineering Research Institute (AGERI), Agriculture Research Centre, Giza, Egypt; 7 Soils and Water Department, Faculty of Agriculture, Benha University, P.O. Box 13736, Moshtohor, Toukh, Qalyoubia Egypt; 8 Department of Soils and Water, Faculty of Agriculture, New Valley University, Kharga, Egypt; 9 National Committee of Soil Sciences, Academy of Scientific Research and Technology, Cairo, Egypt

**Keywords:** Plant sciences, Solid Earth sciences

## Abstract

A comprehensive body of scientific evidence indicates that rhizobial bacteria and melatonin enhance salt tolerance of crop plants. The overall goal of this research was to evaluate the ability of *Rhizobium leguminoserum* bv *phaseoli* to suppress salinity stress impacts in common bean treated with melatonin. Treatments included bacterial inoculations (inoculated (RI) and non-inoculated (NI)), different salinity levels (non-saline (NS), 4 (S1) and 8 (S2) dS m^−1^ of NaCl) and priming (dry (PD), melatonin (PM100) and hydro (PH) priming) with six replications in growing media containing sterile sand and perlite (1:1). The results showed that the bacterial strain had the ability to produce indole acetic acid (IAA), ACC deaminase and siderophore. Plants exposed to salinity stress indicated a significant decline in growth, yield, yield components, nitrogen fixation and selective transport (ST), while showed a significant increase in sodium uptake. However, the combination of PM100 and RI treatments by improving growth, photosynthesis rate and nitrogen fixation positively influenced plant performance in saline conditions. The combined treatment declined the negative impacts of salinity by improving the potassium translocation, potassium to sodium ratio in the shoot and root and ST. In conclusion, the combination of melatonin and ACC deaminase producing rhizobium mitigated the negative effects of salinity. This result is attributed to the increased ST and decreased sodium uptake, which significantly reduced the accumulation of sodium ions in shoot.

## Introduction

Common bean (*Phaseolus vulgaris* L.) is a main grain legume belonging to the Fabaceae family, which includes about 20,000 species^1^. This plant is one of the oldest domesticated crops widely cultivated in cropping systems of many parts of the world due to its ability to fix atmospheric nitrogen^2,3^. The N_2_-fixing legumes have been used as cheaper and more sustainable alternatives to chemical N fertilizers in small-scale farming systems^4^. However, N_2_ fixation can be greatly affected by salinity stress, especially at the initial phases of growth^5^. Hence, N_2_ fixation is usually used as a surrogate to identify salinity tolerance of common bean^6^.

Salinity stress is a major factor limiting crop production worldwide, and is a serious risk to the growth and yield of legume plants in specific^7,8^. In addition, salinity inhibits the uptake and transportation of potassium in plants which in turn causes ion imbalance and toxicity^9^. Among the suggested strategies to combat ionic stress imposed by salinity, combined application of ACC deaminase rhizobium and melatonin has been proposed as a biostimulation strategy^10^.

PGPR’s especially rhizobium bacteria are one of the most important microorganisms in the soil contributing to plant productivity under normal and stress conditions through siderophore production, nitrogen fixation activity, phytohormone biosynthesis, and the biosynthesis of ACC-deaminase^6,11,12^. PGPR’s can enhance the growth of root by producing ACC deaminase and IAA, and by reducing ethylene production in the roots^13^. The potential of *Rhizobium* to enhance growth of plant under stress conditions has been shown in various legume plants^5,14^. Also, symbiotic beneficial rhizobacteria through influencing the bio-synthesis of the proteins having role in cellular defense mechanism, protect the host plants against salt toxicity^6^.

Melatonin (*N*-acetyl-5-methoxytryptamine), an effective biostimulant derived from the amino acid tryptophan, enhances the stress tolerance of plants and bacteria^15–18^. The positive effects of melatonin in alleviating salt stress through scavenging the reactive oxygen species (ROS), maintaining sodium/potassium homeostasis, increasing potassium/sodium ratio, promoting the retention of potassium in roots, and enhancing potassium uptake transporters in the root tips have been well documented^19–21^. In addition, melatonin priming not only improves plant growth and yield but also enhances seed quality^22^. ElSayed et al.^23^ stated that an exogenous application of melatonin regulated the redox homeostasis by its ability to induce either enzymatic or non-enzymatic antioxidant systems in peanut. The impacts of PGPR’s on the development and yield of legumes have been investigated under different environmental stresses, but there is no information on their application with melatonin on legumes in saline conditions^20,21^.

We hypothesized in this study that the cumulative effects of PGPR’s and melatonin will enhance the stress tolerance of common bean grown under saline conditions, probably by modulating the stress-responsive pathways. Hence, the research specific objectives were: (1) to investigate some PGP traits of *Rhizobium leguminosarum* b.v. *phaseoli*; and (2) explore the interactive effect of melatonin priming and PGPR to suppress salinity stress by enhancing ion homeostasis and nitrogen fixation in common bean. Such knowledge enables us to use the potential of PGPR in combination with melatonin priming to improve plant tolerance to salinity.

## Materials and methods

### Rhizobium tests

All methods were performed in accordance with the relevant guidelines and legislations. The bacteria *Rhizobium leguminosarum* b.v. *phaseoli* was obtained from the Department of Soil Sciences, Shiraz University. This bacterium was selected based on its traits of PGP such as IAA production, siderophore production and ACC deaminase activity. IAA production, siderophore production and ACC deaminase activity were calculated by following the methods of Dell’Amico et al.^24^, Glickmann and Dessauxm^25^ and Alexander and Zuberer^26^, respectively.

### Plant tests

#### Experimental details

A greenhouse pot experiment was undertaken at Shiraz University, Shiraz from May to September of 2019. This experiment was carried out in a factorial arrangement based on a completely randomized design (CRD) with six replicates to test the effects of three factors i.e. : (1) salinity stress (non-saline (NS), 4 dS m^−1^ (S1), and 8 dS m^−1^ (S2)) (2) priming treatments (PH (hydro priming), PM100 (melatonin priming (100 µM)) and PD (no priming)) and (3) bacterial inoculations (non-inoculated (NI), and inoculated (RI)).

#### Soil characteristics

The sandy soil (0–20 cm in depth) was collected from the Bakhtegan Lake in Fars, Iran (29° 16′ N; 53° 52′ E). The sandy soil had organic matter content of 1400 mg kg^−1^, available P of 5.5 mg kg^−1^, available K of 65.56 mg kg^−1^, total nitrogen of 530 mg kg^−1^ and available NO_3_^–1^ of 4.53 mg kg^−1^. Its electrical conductivity (EC) and the hydrogen potential (pH) were 0.89 dS m^−1^ and 7.62, respectively. The sand was sterilized by autoclaving, and around 6 kg of sand and perlite (1:1, v/v) mixture were mixed into PVC pots (diameter = 15 cm, height = 60 cm).

#### Priming treatments

Common bean seed priming was done at different concentrations (PD, PH, 20, 100, and 500 µM melatonin) as previously reported^27^. Based on the results, 100 µM melatonin for a duration of 10 h had the highest effect on germination indices compared to the other concentrations^27^.

#### Preparation of rhizobium

Bacterial inoculum was prepared by cultivating the bacterial strain in Yeast Mannitol Broth (YMB) medium^28^, followed by incubation at 27 °C on a rotary shaker (105 rpm) for two days. The bacterial cells were centrifuged at 5000 rpm for 5 min and the pellet was washed with sterile phosphate buffered saline and repeating centrifugation steps. The bacterial pellets diluted with PBS solution to make a final concentration of 10^8^ cells/mL. Then, 2 ml of the bacterial suspension was pipetted into the soil surrounding the seed; 2 ml of sterile PSB solution was added to the NI.

#### Growth conditions and treatments

Common bean seeds (cv. Dorsa) were obtained from Markazi Agricultural and Natural Resources Research and Education Center, Iran. The seeds were surface-sterilized using NaClO solution (3%) and then washed twice by distilled water. Sterilized seeds were primed and sown in Petri-dishes and were misted with distilled water. Seeds started germination one day after sowing, and reached about 80% germination two days later. Thereafter, four uniform seedlings were inoculated and transferred to 15-cm diameter disinfected PVC pots containing autoclave-sterilized substrate (50% perlite and 50% sand). Pots were watered daily with a nitrogen-free nutrient solution according to Broughton and Dilworth^29^. Upon emergence of the first trifoliolate leaf, NaCl was added to the irrigation water to apply salinity treatments: 4 and 8 dS m^−1^ (using 2.6, and 5.1 g L^−1^ NaCl in tap water, respectively)^30^. Plants were thinned to two healthy plants at the four-leaf stage.

#### Measurement of growth, yield and yield components

At the physiological maturity stage, plants were harvested i.e. shoots, nodules, height, number of pod, grain number, pod yield, thousand-grain weight, grain yield, root length and total number of nodules were measured on both plants in each pot. The shoot and root biomass were determined after drying plants at 70ºC until constant weight.

#### Total chlorophyll and net photosynthesis rate measurement

The chlorophyll contents of common bean leaves were measured by the Arnon^31^ method. In short, fresh leaves (0.1 g) were extracted in 80% acetone (10 mL), and then the extract was centrifuged for five minutes at 300 rpm. The supernatant was separated and used for the measurement of chlorophyll contents at 646, and 663 nm using an ultraviolet–visible spectrophotometer (7315 UV/visible Spectrophotometer, Jenway, UK)^32^.

Forty days after sowing, net photosynthesis rate (Pn) of fully developed leaves (six replicates) was recorded using a portable LCi photosynthesis system (ADC Bioscientific Ltd., UK) between 9:00 and 11:00 AM. The system was calibrated before data recording.

#### Measurements of amount of fixed nitrogen and protein content

After harvesting, oven-dried (48 h at 70 °C) shoot and root samples—from each treatment were powdered using an electric mill. The shoot and root nitrogen concentration was determined using titration after distillation by the Kjeldahl method^33^. Total nitrogen (N) content per plant, the N fixed and the crude protein were calculated following the methods of Yaman and Cinsoy^34^ and Egan et al.^35^, respectively.

$$N \,fixed=\text{Plant N content in inoculated pots}-\text{plant N content in uninoculated pots}$$

#### Tissue ion accumulation

Dry material (0.5 g) was taken and ashed at 500 °C for 5 h. After complete dissolution in 2 N HCl, the mixture was filtered using filter paper. The final volume of 50 mL in the flask was reached by adding deionized water. The ionic concentrations ‏were determined using a flame photometer (Sherwood Science, United Kingdom)^36^.

The ionic translocation, sodium uptake, and selective transport (ST) were determined using the equations given by Wang et al.^37^, Shahzad^38^ and Yan et al.^39^, respectively.

$${Na}^{+}\text{translocation factor}=\frac{{Na}^{+ }concentration \,in \,shoots}{{Na}^{+} \,concentration \,in \,roots}$$

$${K}^{+}\text{translocation factor}=\frac{{K}^{+ }concentration \,in \,shoots}{{K}^{+} \,concentration \,in \,roots}$$

$${Na}^{+}\text{uptake at root surface}=\frac{{Total Na}^{+ }concentration \,in \,common \,bean \,plant}{Root \,dry \,weight}$$

$$\text{ST}=\frac{{Na}^{+}{/K}^{+}in \,roots}{{Na}^{+}{/K}^{+}in \,shoots}$$

#### Determination of electrical conductivity (EC) and potential of hydrogen (pH)

At the end of the experiment, 100 g of sand were collected, dried and mixed with deionized water (100 mL) at 25 °C for 24 h. Soil pH (1:2 soil/water suspension) was measured using a pre-calibrated pH-meter (HI 2211 pH/ORP Meter, HANNA Instruments, Woonsocket, RL, USA). EC in soil saturation extracts was determined using a pre-calibrated EC-meter (Hanna HI-2315 Bench Top Conductivity Meter, Hanna Instruments Ltd, USA).

### Statistical analysis

One-way analysis of variance (ANOVA) for the factorial experiment based on a CRD was performed in six replicates, using SAS v. 9.1 software^40^. Mean comparisons were made between treatments through the LSD test at 5% probability level. In addition, regression analysis was performed using Excel software to estimate the relations between traits.

## Results

### Rhizobium tests

**Figure 1:**
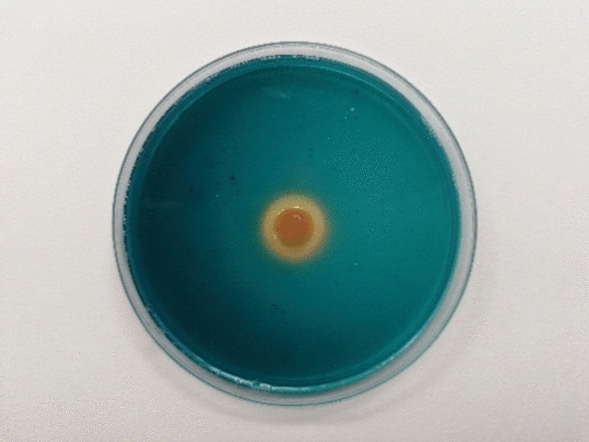
The appearance of orange color and zone formation indicating siderophore production by *Rhizobium leguminosarum* in CAS agar plate assay.

The bacterial strain was investigated for the production of PGP traits i.e. IAA, ACC deaminase and siderophore which are indicated in Table 1 and Fig. 1. The IAA production by the bacteria increased to 8.45 μg ml^*−*1^ in the presence of L-tryptophan in the media.

**Table 1:** *Rhizobium leguminosarum* strain traits.

Strain	IAA (μg mL^−1^)	ACC deaminase (optical density)			Siderophore	
		DF + (NH_4_)_2_SO_4_^a^	DF + ACC^b^	DF^c^	Halo diameter (cm)	(Halo diameter to colony diameter ratio)
*Rhizobium leguminosarum*	8.45	1.706	0.534	0.043	1.83	1.99

DF + (NH_4_)_2_SO_4_^a^: positive control with (NH_4_)_2_SO_4_. DF + ACC^b^: selective medium with 1-aminocyclopropane-1-carboxylate. ^c^DF: negative control (DF).

*Rhizobium leguminoserum bv phaseoli* was able to grow on DF minimal medium containing ACC as the sole N source, indicating that they have the ACC deaminase activity. Optical densities of *Rhizobium leguminosarum* suspensions in minimal medium without nitrogen source (negative control), amended with 2 g of (NH_4_)_2_SO_4_ L^−1^ (positive control), or with ACC (3 mmol L^−1^) are listed in Table 1.

### Plant tests

#### Growth parameters

**Figure 2:**
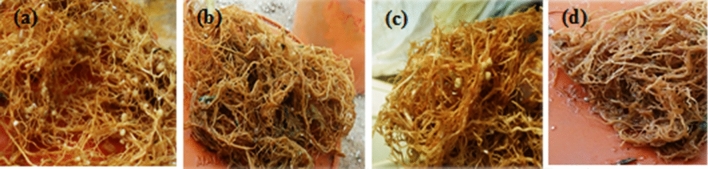
Images depicting the nodulation of common bean plants under non-saline conditions (**a**) RI + PM100; (**b**) NI + PM100; (**c**) RI + PD; (**d**) NI + PD. NI and RI indicate non-inoculated and *Rhizobium* inoculated plants, respectively. PM100 and PD represent melatonin priming and non-priming, respectively.

With enhancing salinity levels, height, shoot and root biomass, and root length decreased. However, salinity appears to affect shoots more than roots (Table 2). The melatonin application, regardless of salinity level and RI, improved height, shoot and root biomass, root length, and number of nodules compared to their respective PD treatments by 68.2%, 43.7%, 38.2%, 5.5%, and 8.9 times, respectively (Table 2). However, application of S2, regardless of the priming and inoculation treatments, led to a decrease of 79.5% in the height, 27.1% in shoot biomass, 24.4% in root biomass, and 70.5% in the number of nodules compared to NS (Table 2). Height, shoot and root biomass, and length of root, improved when PM100 was applied with RI (Table 2). The combination of PM100 and RI treatments increased height, shoot biomass, root length and root biomass by 11.6%, 13.5%, 8.4% and 7.5%, respectively (Table 2). Of the bacterial inoculated plants, those primed with melatonin achieved the highest total number of nodules (332.25) under NS level (Table 2; Fig. 2a–d).

**Table 2:** The effects of bacterial inoculation, salinity and priming on height, shoot and root biomass, root length and the nodules number of common bean.

Factor	Height (cm)	Shoot biomass (g per pot)	Length of root (cm)	Root biomass (g per pot)	Total number of nodules per pot
**Bacterial inoculations (I)**					
NI	49.38^b^	9.73^b^	32.83^b^	2.75^b^	0^b^
RI	66.27^a^	11.58^a^	36.12^a^	3.21^a^	113.80^a^
**Salinity levels (S)**					
NS	110.50^a^	12.36^a^	40.23^a^	3.40^a^	92.45^a^
S1	40.33^b^	10.60^b^	37.07^b^	2.96^b^	50.95^b^
S2	22.66^c^	9.01^c^	26.13^c^	2.57^c^	27.29^c^
**Priming (P)**					
PM100	74.00^a^	12.36^a^	36.92^a^	3.40^a^	101.79^a^
PH	55.50^b^	11.01^b^	34.14^b^	3.07^b^	57.45^b^
PD	44.00^c^	8.60^c^	32.36^c^	2.46^c^	11.45^c^
**Bacterial inoculations × Salinity levels × Priming (I × S × P)**					
**NI**					
**NS**					
PM100	120.50^b^	13.74^a–e^	46.25^b^	4.01^ab^	0^j^
PH	105.50^c^	15.08^a–c^	45.01^c^	3.01^e^	0^j^
PD	95.50^e^	10.42^fg^	41.05^e^	2.51^g^	0^j^
**S1**					
PM100	31.50^i^	12.02^c–g^	39.88^f^	2.91^ef^	0^j^
PH	21.50^k^	12.02^c–g^	32.86^i^	2.91^ef^	0^j^
PD	17.50^lm^	10.42^fg^	27.35^j^	2.11^h^	0^j^
**S2**					
PM100	23.50^j^	10.94^e–g^	25.92^k^	2.51^g^	0^j^
PH	16.50^m^	10.68^e–g^	24.91^l^	2.51^g^	0^j^
PD	12.50^n^	8.82^g^	24.80^l^	2.01^h^	0^j^
**RI**					
**NS**					
PM100	134.50^a^	15.6^ab^	50.13^a^	4.31^a^	332.25^a^
PH	106.50^c^	16.6^a^	46.35^b^	3.56^cd^	194.25^b^
PD	100.50^d^	12.42^b–f^	46.02^b^	3.01^e^	28.25^h^
**S1**					
PM100	86.50^f^	14.54^a–d^	44.08^d^	3.81^bc^	150.25^c^
PH	64.50^g^	15.08^a–c^	37.18^g^	3.41^d^	120.25^e^
PD	20.50^k^	10.82^e–g^	34.97^h^	2.61^fg^	35.25^f^
**S2**					
PM100	47.50^h^	11.6^d–g^	26.97^j^	2.86^e–g^	128.25^d^
PH	18.50^l^	11.7^d–g^	26.87^j^	2.78^e–g^	30.25^g^
PD	–	–	–	–	–
**Analysis of variance**					
I	***	***	***	***	***
S	***	***	***	***	***
P	***	***	***	***	***
I × S	***	ns	***	ns	***
I × P	***	ns	***	ns	***
S × P	***	***	***	***	***
I × S × P	***	***	***	***	***

I, S and P are bacterial inoculations, salinity levels and priming treatments. NI and RI are non-inoculated and *Rhizobium* inoculated plants. NS, S1 and S2 are non-saline, 4 and 8 dSm^−1^ of salinity stress, respectively. PM100, PH and PD are melatonin priming, hydro priming and non-priming, respectively. ns, *, ** and *** indicate non-significant and significance at 5%, 1% and 0.1%, respectively. Different letters within a column indicate means that are significantly different at P < 0.05.

#### Total chlorophyll and net photosynthesis rate (Pn) measurement

**Figure 3:**
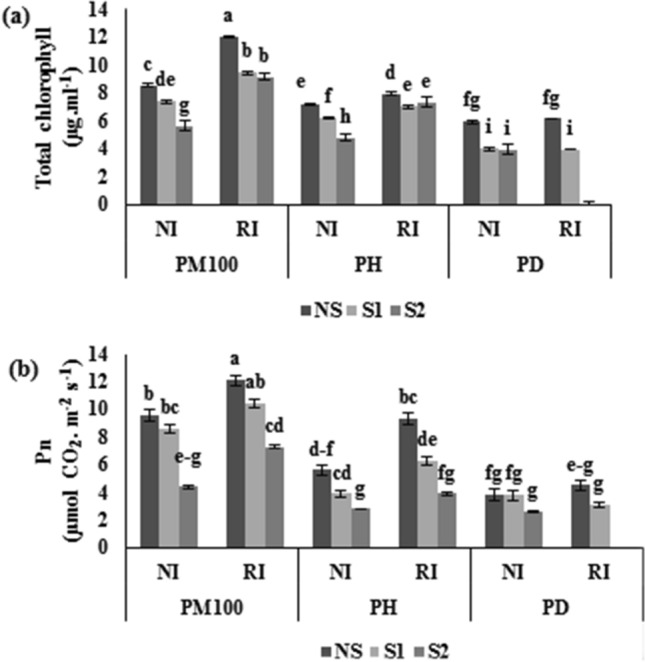
Effects of salinity stress, ACC deaminase-producing rhizobial bacterium and melatonin on chlorophyll (**a**) and Pn (**b**) of common bean. PM100, melatonin priming; PH, hydro priming; PD, no priming; NI, non-inoculated; RI, *Rhizobium* inoculated; NS, S1 and S2 are non-saline, 4 and 8 dSm^−1^ of salinity stress, respectively. The vertical bars followed the different letters were significantly differences according to the LSD test at P < 0.05.

The regular exposure of plants to salinity stress decreased the total chlorophyll concentration and Pn. The results showed that the PM100 plus RI treatments significantly improved the total chlorophyll as compared to the PD plus NI treatments (Fig. 3a,b). Under NS condition, the combination of RI and PM100 treatments increased total chlorophyll and Pn compared to the PD plus NI treatments by 3.04 and 4.65 times, respectively (Fig. 3a,b).

#### Nitrogen concentration and content, protein content and amount of fixed nitrogen

The results presented in Table 3 show that nitrogen concentration, nitrogen content and shoots and roots protein content under salinity stress conditions were lower than those under normal conditions. The plants inoculated with RI or primed with melatonin showed a significant increase in all parameters compared to NI or PD plants, irrespective of the salinity treatment. The interaction of RIS and P treatments resulted in the maximum increase of all tested parameters in plants primed with melatonin and inoculated with the bacterial isolate, compared to those tested alone in NS, S1 and S2 (Table 3). Under salinity condition (S1), nitrogen concentration and content in the shoots and roots enhanced significantly (2.1, 1.6, 2.8, and 2.1 times, respectively) in RI + PM100 treatment compared to NI + PM100 (Table 3). The shoots and roots protein content of treated with RI and PM100 significantly increased by 2.1, 1.6, 1.3 and 1.3 times, respectively, compared to the NI under salinity conditions (S1 and S2) (Table 3). On the other hand, the stressed and non-stressed plants primed with melatonin and inoculated with *Rhizobium* had significantly higher values of biochemical parameters; nitrogen concentration and content, and protein content in shoots and roost, compared to non-treated ones.

**Table 3:** The effects of bacterial inoculation, salinity levels and melatonin on nitrogen concentration and content, and content of protein in shoots and roots of common bean.

Factor	Nitrogen concentration (%)		Nitrogen content (g pot^−1^)		Protein content (%)	
	Shoot	Root	Shoot	Root	Shoot	Root
**Bacterial inoculations (I)**						
NI	1.42^b^	1.40^b^	14.14^b^	3.99^b^	8.87^b^	8.76^b^
RI	2.13^a^	1.69^a^	25.25^a^	5.58^a^	13.33^a^	10.60^a^
**Salinity levels (S)**						
NS	2.08^a^	1.73^a^	26.63^a^	6.12^a^	13.02^a^	10.87^a^
S1	1.70^b^	1.57^b^	18.64^b^	4.79^b^	10.64^b^	9.85^b^
S2	1.54^c^	1.33^c^	13.83^c^	3.44^c^	9.63^c^	8.32^c^
**Priming (P)**						
PM100	2.10^a^	1.68^a^	27.10^a^	6.02^a^	13.17^a^	10.52^a^
PH	1.56^b^	1.62^a^	17.55^b^	5.03^b^	9.75^b^	10.13^a^
PD	1.66^b^	1.34^b^	14.45^c^	3.30^c^	10.38^b^	8.39^b^
**Bacterial inoculations × Salinity levels × Priming (I × S × P)**						
**NI**						
**NS**						
PM100	1.43^g–j^	1.75^c^	21.22^de^	7.04^c^	8.98^g–j^	10.94^c^
PH	1.82^d–g^	1.56^de^	19.60^d–g^	4.72^e^	11.39^d–g^	9.77^de^
PD	1.62^f–i^	1.38^e–g^	14.30^h–j^	3.48^g–j^	10.18^f–i^	8.66^e–g^
**S1**						
PM100	1.21^jk^	1.26^gh^	12.60^i–l^	3.69^f–i^	7.56^jk^	7.90^gh^
PH	1.52^g–j^	1.54^e^	15.85^f–i^	4.50^ef^	9.54^g–j^	9.64^e^
PD	1.34^ij^	1.49^ef^	9.60^kl^	3.18^h–j^	8.38^ij^	9.35^ef^
**S2**						
PM100	1.72^e–i^	1.12^h^	15.17^g–j^	2.84^ij^	10.80^e–i^	7.03^h^
PH	0.86^k^	1.34^fg^	8.20^l^	3.73^f–h^	5.37^k^	8.40^fg^
PD	1.22^jk^	1.14^h^	10.74^j–l^	2.69^j^	7.63^jk^	7.13^h^
**RI**						
**NS**						
PM100	3.42^a^	2.37^a^	54.80^a^	10.26^a^	21.42^a^	14.84^a^
PH	2.14^b–d^	2.00^b^	27.79^c^	7.17^bc^	13.39^b–d^	12.55^b^
PD	2.04^c–e^	1.35^fg^	22.05^de^	4.07^e–g^	12.77^c–e^	8.43^fg^
**S1**						
PM100	2.52^b^	2.07^b^	35.25^b^	7.94^b^	15.75^b^	12.98^b^
PH	1.64^f–i^	1.72^cd^	20.37^d–f^	5.90^d^	10.28^f–i^	10.80^cd^
PD	1.97^c–f^	1.34^fg^	18.17^e–h^	3.52^g–j^	12.34^c–f^	8.42^fg^
**S2**						
PM100	2.31^bc^	1.50^ef^	23.58^cd^	4.34^e–g^	14.49^bc^	9.43^ef^
PH	1.36^h–j^	1.53^e^	13.47^i–k^	4.16^e–g^	8.55^h–j^	9.60^e^
PD	–	–	–	–	–	–
**Analysis of variance**						
I	***	***	***	***	***	***
S	***	***	***	***	***	***
P	***	***	***	***	***	***
I × S	ns	ns	***	***	ns	ns
I × P	***	***	***	***	***	***
S × P	***	***	***	***	***	***
I × S × P	***	***	***	*	***	***

I, S and P are bacterial inoculations, salinity levels and priming treatments. NI and RI are non-inoculation and *Rhizobium* inoculated plants. NS, S1 and S2 are non-saline, 4 and 8 dSm^−1^ of salinity stress, respectively. PM100, PH and PD are melatonin priming, hydro priming and non-priming, respectively. ns, *, ** and *** indicate non-significant and significance at 5%, 1% and 0.1%, respectively. Different letters within a column indicate means that are significantly different at P < 0.05.

**Figure 4:**
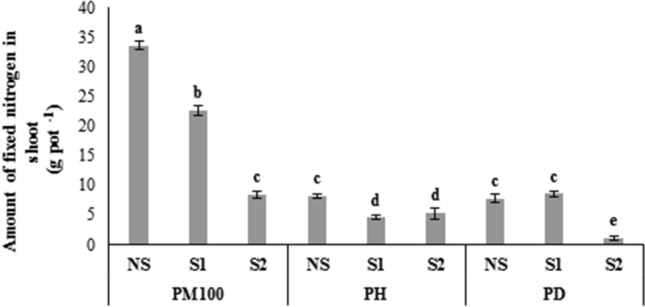
Effects of salinity stress, and melatonin on fixed nitrogen amount in shoot of common bean. PM100, melatonin priming; PH, hydro priming; PD, no priming; NS, S1 and S2 are non-saline, 4 and 8 dSm^−1^ of salinity stress, respectively. The vertical bars followed the different letters were significantly differences according to the LSD test at P < 0.05.

Significant increases in the amount of nitrogen fixation was observed in the PM100 plus NS treatments (33.6 g pot^−1^), followed by the PM100 plus S1 (22.6 g pot^−1^) (Fig. 4). In contrast, the amount of nitrogen fixation decreased by 87.4 and 79.9% in plants that germinated from PD-treated seeds compared to PM100 and PH in the S2 treatment (Fig. 4).

#### Potassium (K^+^), sodium (Na^+^), K^+^/Na^+^ ratio

As shown in Table 4, the concentration of sodium increased significantly in both shoots and roots in the P plus RIS treatments under salinity stress. However, the Na^**+**^ concentration in shoots was 1.8 times lower in the combination of RI, S2 and PM100 treatments than in the combination of NI, S2 and PD, whereas in roots, the Na^**+**^ concentrations was 1.1 times higher. In contrast, under salinity stress, K^**+**^ concentration declined in shoots and roots (Table 4). In shoots, the K^**+**^ concentration increased by 1.4 times in combined treatment of RI, S2 and PM100 compared to the combination of NI, S2 and PM100 treatments. In roots, the K^**+**^ concentration increased more (1.5 times) in the combination of RI, S2 and PM100 treatments than combination of NI, S2 and PM100.

**Table 4:** The effects of bacterial inoculation, salinity and melatonin on concentrations of K^+^ and Na^+^ and K^+^/Na^+^ ratios in shoots and roots of common bean.

Factor	Shoot Na^+^ concentration (mg g^−1^)	Root Na^+^ concentration (mg g^−1^)	Shoot K^+^ concentration (mg g^−1^)	Root K^+^ concentration (mg g^−1^)	Shoot K^+^/Na^+^	Root K^+^/Na^+^
**Bacterial inoculations (I)**						
NI	9.11^a^	8.69^a^	18.70^b^	13.62^b^	5.96^b^	3.12^b^
RI	7.01^b^	8.13^b^	24.14^a^	19.07^a^	8.37^a^	4.78^a^
**Salinity levels (S)**						
NS	1.82^c^	2.58^c^	27.75^a^	22.60^a^	17.53^a^	9.27^a^
S1	8.85^b^	8.91^b^	20.75^b^	15.70^b^	2.71^b^	1.81^b^
S2	13.51^a^	13.75^a^	15.75^c^	10.60^c^	1.26^c^	0.77^c^
**Priming (P)**						
PM100	6.66^c^	8.58^a^	27.75^a^	22.68^a^	10.89^a^	5.39^a^
PH	8.01^b^	8.58^a^	19.59^b^	14.51^b^	6.37^b^	3.37^b^
PD	9.51^a^	8.08^b^	16.92^c^	11.85^c^	4.23^c^	3.08^c^
**Bacterial inoculations × Salinity levels × Priming (RI × S × P)**						
**NI**						
**NS**						
PM100	1.22^k^	2^k^	29^c^	24^c^	25.30^a^	11.11^b^
PH	2^j^	3^j^	23^fg^	18^fg^	10.25^c^	5.81^e^
PD	2^j^	3^j^	23^fg^	18^fg^	10.25^c^	5.81^e^
**S1**						
PM100	9^g^	11^e^	22^g^	17^g^	2.45^e^	1.58^gh^
PH	10^f^	10^f^	13^j^	8^j^	1.34^e^	0.86^i–k^
PD	11^e^	8^h^	12^jk^	7^jk^	1.13^e^	0.95^i–k^
**S2**						
PM100	12^d^	12^d^	18^h^	13^h^	1.53^e^	1.13^h–j^
PH	14^b^	13^c^	11^k^	6^k^	0.83^e^	0.51^jk^
PD	18^a^	14^b^	9^l^	4^l^	0.54^e^	0.33^k^
**RI**						
**NS**						
PM100	1^k^	2^k^	35^a^	30^a^	27.40^a^	13.80^a^
PH	1^k^	2^k^	27^d^	22^d^	21.28^b^	10.21^c^
PD	2^j^	2^k^	24^ef^	19^ef^	10.68^c^	8.87^d^
**S1**						
PM100	5^i^	8^h^	32^b^	27^b^	6.16^d^	3.37^f^
PH	7^h^	9^g^	23^fg^	18^fg^	3.25^e^	2.03^g^
PD	9^g^	6^i^	17^h^	12^h^	1.91^e^	2.05^g^
**S2**						
PM100	10^f^	15^a^	25^e^	20^e^	2.50^e^	1.36^hi^
PH	12^d^	13^c^	15^i^	10^i^	1.28^e^	0.81^i–k^
PD	–	–	–	–	–	–
**Analysis of variance**						
I	***	***	***	***	***	***
S	***	***	***	***	***	***
P	***	***	***	***	***	***
I × S	***	***	***	***	**	***
I × P	*	***	***	***	**	ns
S × P	***	***	***	***	***	***
I × S × P	***	***	*	*	**	**

I, S and P are bacterial inoculations, salinity levels and priming treatments. NI and RI are non-inoculation and *Rhizobium* inoculated plants. NS, S1 and S2 are non-saline, 4 and 8 dSm^−1^ of salinity stress, respectively. PM100, PH and PD are melatonin priming, hydro priming and non-priming, respectively. ns, *, ** and *** indicate non-significant and significance at 5%, 1% and 0.1%, respectively. Different letters within a column indicate means that are significantly different at P < 0.05.

The K^+^/Na^+^ ratio increased significantly in both shoots and roots when seeds primed with melatonin and inoculated with RI compared to the combination of NI and PM100 under NS, S1 and S2 salinity levels (Table 4). The increase in potassium concentration and potassium to sodium ratio, and the reduction of sodium concentration in both shoots and roots, resulting from melatonin priming and *Rhizobium* inoculation, were significant under all salinity levels.

#### Ion translocation and Na^+^ uptake and ST measurement

**Figure 5:**
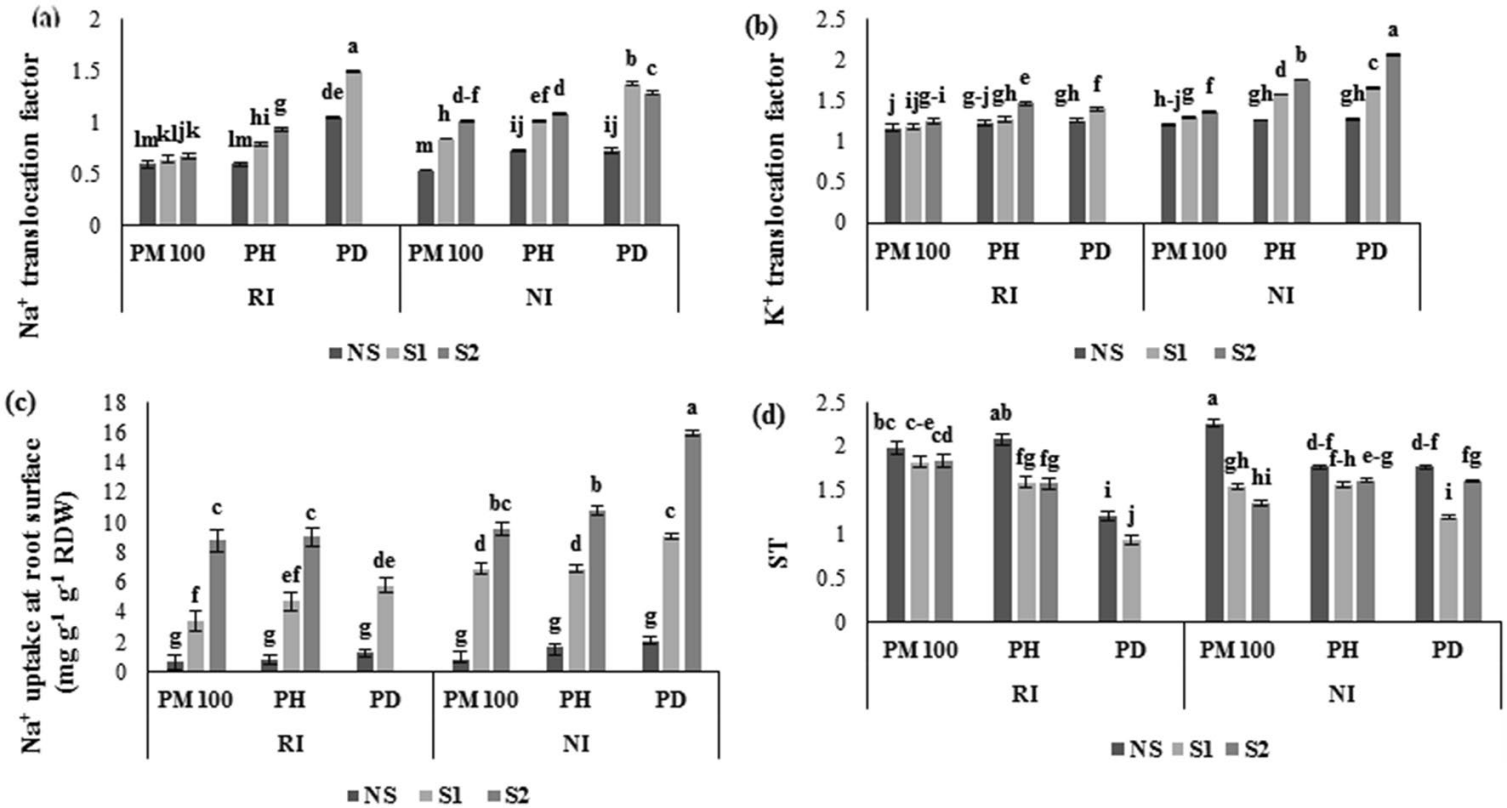
Effects of salinity levels, BI, and priming on Na^+^ and K^+^ translocation factors (**a** and **b**) and selective transport (**c**) of common bean. PM100, priming treatment with 100 µM melatonin; PH, priming treatment with distilled water; PD, dry seed without priming; NI, non- Rhizobium inoculation; RI, Rhizobium inoculation; NS, S1 and S2 are non-saline, 4 and 8 dSm^−1^ of salinity stress, respectively. Data are presented as the means of six replicates; The vertical bars indicate standard deviations. Different letters indicate significant differences according to the LSD test at P < 0.05.

The results shown in Fig. 5a–d presents the changes in potassium, sodium translocation, sodium uptake and ST in the stressed common bean after applying the PM100 plus RI treatments. The common bean seedlings exposed to 4 and 8 dS m^−1^ levels showed that the translocation of sodium and potassium was increased by 90.2 and 77%, and 30.9 and 63.5% in the combined application of PD and NI treatments (Fig. 5a,b), while they increased by 40.1 and 50%, and 38.1 and 24.6% in the combination of PH and NI treatments respectively, as compared to the NS, PD and NI combination treatments (Fig. 5a,b). Conversely, S2 treatment decreased ST by 9%, whereas Na^+^ uptake in roots was also enhanced by 7.98 times in the combination of PD and NI, when compared to the NS level. The combination of PM100 and RI treatments under severe salinity conditions (S2) improved ST (2.2%), while Na^+^ translocation and Na^+^ uptake were reduced by 1.22 and 2.88 times, respectively, compared to combined NS, PD and NI (Fig. 5).

#### Soil EC and pH

**Figure 6:**
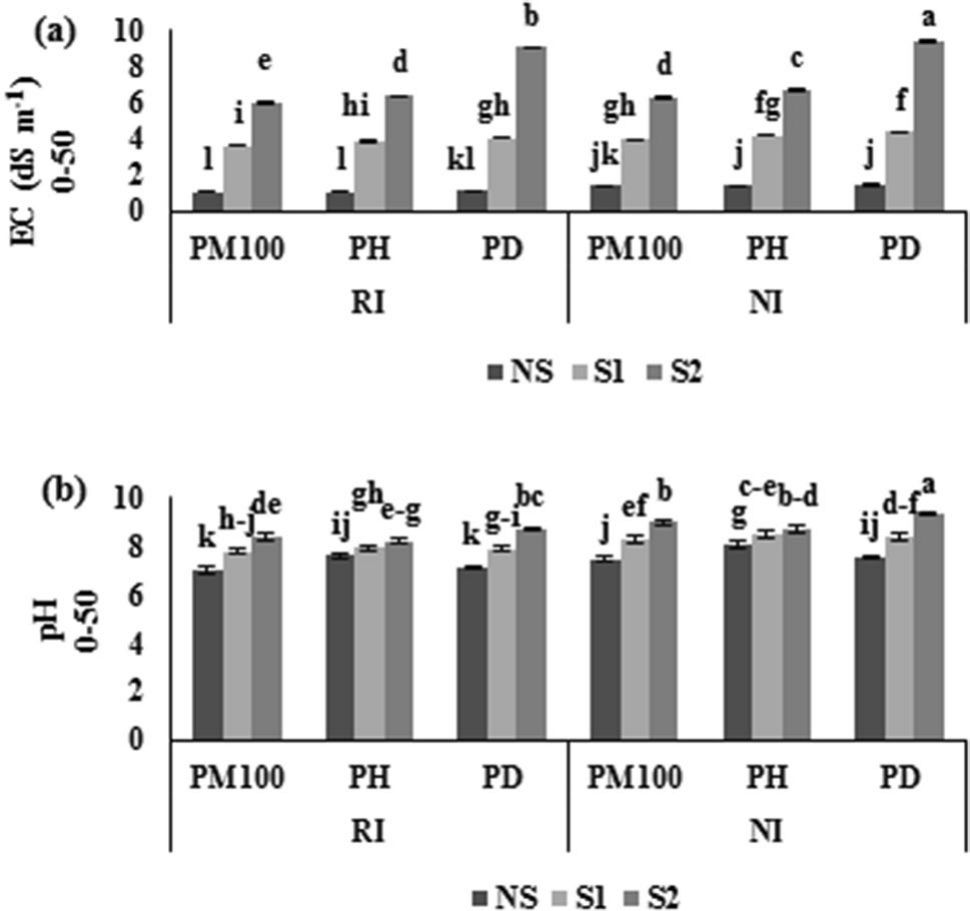
Effects of salinity stress, ACC deaminase-producing rhizobial bacterium and melatonin on EC and pH of soil (**a**) EC of the soil within 0–50 cm depth, (**b**) pH of the soil within 0–50 cm depth. PM100, melatonin priming; PH, hydro priming; PD, no priming; NI, *Rhizobium* non-inoculation; RI, *Rhizobium* inoculation; NS, S1 and S2 are non-saline, 4 and 8 dS m^−1^ of salinity stress, respectively. The vertical bars followed the different letters were significantly differences according to the LSD test at P < 0.05.

Soil EC and pH were highly affected by the combination of RI and PM100 treatments under salinity stress (Fig. 6a,b). The results showed that the PM100 plus RI treatments were significantly decreased EC by 57.6, 20, and 37.4% and pH by 10.6, 7.4, and 8.4% compared to the PD plus NI treatments under NS, S1 and S2 levels (Fig. 6a,b).

#### Yield and yield components

Both P and RI treatments caused significant increases in all yield components (Table S1). The combination of PM100 and RI treatments produced the highest number of pods (9 plant^−1^), pod yield (42.9 g pot^−1^), number of grains (5 pod^−1^), thousand-grain weight (299.2 g) and grain yield (13.8 g pot^−1^), an increase of 71.4, 26.6, 25, 4.55 and 23.4% compared to the PM100 plus NI treatments under NS, respectively. Additionally, under saline conditions, the maximum number of pods (plant^−1^), pod yield, number of grains (pod^−1^), thousand-grain weight and grain yield were found in the combined PM100, RI and S1 treatments, with an average of 7, 17.84 g pot^−1^, 4, 279.2 g, and 12.4 g pot^−1^, representing 2.2, 2.0, 1.3, 1.0 and 1.2 times increases compared to the combined PM100, NI and S1 treatments, respectively. The lowest number of pods (plant^−1^), pod yield, number of grains (pod^−1^), thousand-seed weight and grain yield were recorded for the combined of PD, RI and S2 treatments, where no pod was produced (Table S1).

#### Relationships between amounts of fixed nitrogen, ST and growth parameters

Significant positive polynomial relationships were observed between the amount of nitrogen fixation and the total number of nodules, potassium concentration of root, the potassium to sodium ratio of shoots and roots and ST across the salinity-priming treatments (Supplementary Fig. S1). The K^+^ concentration in both shoots and roots showed significant positive exponential relationships with the amount of fixed nitrogen (Figure S1).

As shown in Figure S2, across all treatments, significant positive linear relationships were observed in the number of pods (plant^−1^), pod yield, number of grains (pod^−1^), grain yield with ST, while significant linear and logarithmic relationships were found in ST for length of root, root biomass and number of nodules (Figure S3).

Furthermore, the relationships between the enhancement rates for the functional traits, such as amount of fixed nitrogen, chlorophyll, and Pn were investigated. As shown in Figure S4, the amount of fixed nitrogen enhancement rate had positive power and exponential relationships with chlorophyll and Pn.

## Discussion

Salinity stress negatively affects plant attributes by influencing ion toxicity and ethylene signaling^41^. Although melatonin and ACC deaminase-producing rhizobium have been implicated in the amelioration of the plant development and protection against salinity in various crops^13,20^, there is no sufficient information on the influence of both in saline conditions. The results showed that the combination of RI and PM100 significantly improved shoot and root growth, and yield in salinity treatments by maintaining ion homeostasis and increasing chlorophyll content and photosynthesis. A significant increase in growth of shoots and roots showed the RI efficacy along with melatonin compared to the RI plus PD treatments in saline conditions.

The negative impact of increased salinity on grain yield and biomass of common bean was significant in the present study. Growth, yield and yield components of the common bean grown under high-salinity level (8 dS m^−1^) were significantly reduced compared to NS condition. In addition, a substantial increase was observed in the growth and yield of plants exposed to RI and PM100. Salinity stress reduced plant biomass where no RI and PM100 were applied due to the reduction of chlorophyll content and photosynthesis. High production of chlorophyllase and degradation of chlorophyll in salinity treatments leads to accelerate leaf senescence^42,43^. The combination of RI and PM100 increased the photosynthetic efficiency by inhibiting the expression of chlorophyllase gene resulting in protecting the photosynthetic pigments from degradation^10,18^. Furthermore, the important role of melatonin as an antioxidant is directly linked with the improvement of plants’ growth parameters and yield. Siddiqui et al.^44^ noted that the inhibition of chlorophyll degradation and improvement in photosynthetic capacity by applying melatonin played a crucial role in the enhancement of growth and yield under stress.

Yan et al.^45^ pointed out that the rate of sodium and potassium uptake was controlled by application of melatonin. Greater amounts of potassium in the shoots of common bean at S1 and S2 levels in RI + PM100 treatment might be another responsible factor for mitigating salinity stress. According to Jiang et al.^20^, high ion homeostasis and decreasing ion toxicity by melatonin contribute to the increased concentration of potassium and potassium/sodium ratio in plants. Better ST can maintain ion homeostasis by compartmentalization of ions into vacuoles and decrease the amount of the ions in cytoplasm^46,47^. Similarly, an increase in potassium concentration in shoots and ST as well as a decreased sodium uptake and translocation were observed in the RI plus melatonin treatments. The increase in potassium concentration, potassium/sodium ratio and ST might also contribute to the higher salt tolerance of RI in the presence of melatonin which in turn finally increases transporters of potassium under S1 and S2. Our findings are in agreement with previous findings of Abd El-Ghany and Attia^10^, who showed that melatonin improves the abiotic stress tolerance of PGPR.

The improvement in the growth of shoots and roots might be also due to a reduction in ethylene levels by *Rhizobium leguminosarum* b.v. *phaseoli*. Production of ACC-deaminase by this bacterium may be the crucial cause of plant better tolerance than NI in saline conditions. Dubois et al.^47^ found an increase in the production of ethylene in roots and shoots in saline conditions by increasing the ACC level in plants. The released ACC from roots into the rhizosphere is hydrolyzed by ACC-deaminase into ammonia and α-ketobutyrate which ultimately reduces ethylene level^48^. Reduction in ethylene will lead to a greater growth of roots which will increase plant nutrients uptake by increasing root surfaces^46^. *Rhizobium leguminosarum* b.v. *phaseoli* reduced the negative impacts of salinity in common bean in terms of enhancement in net photosynthesis rate and total chlorophyll content.

In addition to the ACC-deaminase, the improvement in common bean growth might also be favored by the production of siderphore and IAA by *Rhizobium leguminosarum* as compared to NI. Benková et al.^49^ indicated enhancement in the control of cell division, differentiation and elongation induced by IAA synthesis by PGPR. These plant physiological processes are crucial for organ initiation and differentiation such as nodule development. The IAA produced by PGPR increases free amino acids in host plants that play an important role in photosynthetic products supplied to nodules^8,50^. In addition, increases the expression of the genes involved in carbon transport to bacteroids^50,51^. Additionally, siderphores synthesis and organic acids production by PGPR increase nutrient availability via the enhancement of chelate potassium ions, and change the EC and pH of the rhizosphere^52,53^.

## Conclusion

Salinity stress is one of the most serious limiting factors of common bean production that causes changes in transportability of sodium and potassium ions and nitrogen fixation. It is concluded that both melatonin and RI effectively decline the severity of stress in common bean by reducing the effects of stress ethylene and increasing selective transportability for sodium and potassium ions and nitrogen fixation activity. The combination of melatonin and RI is a better approach than individual application of melatonin and RI under salinity stress for improving growth, yield and yield components and mitigating salinity toxicity in common bean. More studies are required to introduce the halotolerant PGPR combined with melatonin as an efficient treatment to alleviate salinity in common bean and subsequently improve salt tolerance of this crop under field conditions.

## Supplementary Information

Supplementary Information.

## Data Availability

The datasets generated during and/or analyzed during the current study are available from the corresponding author on reasonable request.
