# Supplementary material for: Co-application of ACC deaminase-producing rhizobial bacteria and melatonin improves salt tolerance in common bean (Phaseolus vulgaris L.) through ion homeostasis
Source: Sci Rep. 2022 Dec 21;12:22105. doi: 10.1038/s41598-022-26084-3 (PMC9772384; doi:10.1038/s41598-022-26084-3)
Supplement: Supplementary file 1 — Supplementary Information. [file 41598_2022_26084_MOESM1_ESM.docx]

**Co-application of ACC deaminase-producing rhizobial bacteria and melatonin improves salt tolerance in common bean (*Phaseolus vulgaris* L.) through [ion homeostasis](https://www.sciencedirect.com/topics/neuroscience/ion-homeostasis)**

Mozhgan Alinia^1^, Seyed A. Kazemeini^1*^, Ali Dadkhodaie^1^, Mozhgan Sepehri^2^, Vahid A. Jahandideh Mahjenabadi ^3^, Syeda F. Amjad^4*^ Peter Poczai^5*^, Doaa El-Ghareeb^6^, Mohamed A. Bassouny^7^, Ahmed A. Abdelhafez^8,9*^

**^1^** Former PhD Student of Plant Production and Genetics Department, Shiraz University, Shiraz, Iran. [mozhgan.alinia@gmail.com](mailto:mozhgan.alinia@gmail.com)

**^1^** Professor of Plant Production and Genetics Department, Shiraz University, Shiraz, Iran. [akazemeini@shirazu.ac.ir](mailto:akazemeini@shirazu.ac.ir)

**^1^** Associate Professor of Plant Production and Genetics Department, Shiraz University, Shiraz, Iran. [dadkhodaie@shirazu.ac.ir](mailto:dadkhodaie@shirazu.ac.ir)

^2^Assistant Professor of Soil Science Department, Shiraz University, Shiraz, Iran. [m.sepehri@shirazu.ac.ir](mailto:m.sepehri@shirazu.ac.ir)

**^3^** Researcher of Agriculture Research, Education and Extension Organization, Soil and Water Research Institute, Karaj, Iran. [vahid.jahandideh67@gmail.com](mailto:vahid.jahandideh67@gmail.com)

^4^ Department of Botany, University of Agriculture Faisalabad, Pakistan. [fasihamushadi75@gmail.com](mailto:fasihamushadi75@gmail.com)

^5^ Botany Unit, Finnish Museum of Natural History, University of Helsinki, FI-00014 Helsinki, Finland. [peter.poczai@helsinki.fi](mailto:peter.poczai@helsinki.fi)

^6^ Agriculture Genetic Engineering Research Institute (AGERI), Agriculture Research Centre, Egypt. [dekeshek@uqu.edu.sa](mailto:dekeshek@uqu.edu.sa)

^7^ Soils and Water Department, Faculty of Agriculture, Benha University, Moshtohor, Toukh, Qalyoubia, P.O. Box 13736, Egypt. [mohamed.bassuony@fagr.bu.edu.eg](mailto:mohamed.bassuony@fagr.bu.edu.eg)

^8^ Department of Soils and Water, Faculty of Agriculture, New Valley University, Egypt.

^9^ National Committee of Soil Sciences, Academy of Scientific Research and Technology, Egypt. [ahmed.aziz@agr.nvu.edu.eg](mailto:ahmed.aziz@agr.nvu.edu.eg)

Correspondence:

Ahmed A. Abdelhafez: [ahmed.aziz@agr.nvu.edu.eg](mailto:ahmed.aziz@agr.nvu.edu.eg)

Seyed A. Kazemeini: [akazemeini@shirazu.ac.ir](mailto:akazemeini@shirazu.ac.ir)

Syeda F. Amjad: [fasihamushadi75@gmail.com](mailto:fasihamushadi75@gmail.com)

Peter Poczai: [peter.poczai@helsinki.fi](mailto:peter.poczai@helsinki.fi)

|  |  |
| --- | --- |
|  |  |
|  |  |
|  | |
| **Figure S1.** Relationships between amount of fixed nitrogen and root biomass (a), total number of nodules (b), K^+^ concentration in shoots and roots (c and d), K^+^/Na^+^ ratio in shoots and roots (e and f), selectivity transport (ST) (g) of common bean plants affected by salinity levels, I and priming treatments. ns, *, ** and *** indicate non-significant and significance at 5%, 1% and 0.1%, respectively. | |

|  |  |
| --- | --- |
|  |  |
| **Figure S2.** Relationships between number of pods per plant (a), pod yield (b), grain number per pod (c), grain yield (d) and selective transport of common bean plants affected by salinity levels, I and priming treatments. ns, *, ** and *** indicate non-significant and significance at 5%, 1% and 0.1%, respectively. | |

| **(a)** |  |
| --- | --- |
|  | |
| **Figure S3.** Relationships between selective transport and, root length (a), root biomass (b) total number nodules per pot (c) of common bean plants affected by salinity levels, I and priming treatments. ns, *, ** and *** indicate non-significant and significance at 5%, 1% and 0.1%, respectively. | |

|  |  |
| --- | --- |
| **Figure S4.** Relationships between amount of fixed nitrogen, chlorophyll (a) and Pn (b) of common bean. plants affected by salinity levels, I and priming treatments. ns, *, ** and *** indicate non-significant and significance at 5%, 1% and 0.1%, respectively. | |

| **Table S1**. The effects of bacterial inoculation, salinity levels and priming on yield and yield components of common bean. | | | | | | | |
| --- | --- | --- | --- | --- | --- | --- | --- |
| Grain yield  (g pot^-1^) | 1000-grain weight (g) | Grain number per pod | Pod yield  (g pot^-1^) | Pod number per plant | Factor | | |
|  |  |  |  |  | Bacterial inoculations (I) | | |
| 7.75^b^ | 198.63^b^ | 2.05^b^ | 13.19^b^ | 2.58^b^ | NI | | |
| 10.50^a^ | 210.17^a^ | 2.88^a^ | 21.19^a^ | 5.66^a^ | RI | | |
|  |  |  |  | Salinity levels (S) | | | |
| 10.99^a^ | 264.09^a^ | 3.50^a^ | 35.19^a^ | 6.12^a^ | NS | | |
| 9.64^b^ | 245.34^b^ | 2.20^b^ | 11.19^b^ | 4.12^b^ | S1 | | |
| 6.74^c^ | 103.77^c^ | 1.70^c^ | 5.18^c^ | 2.12^c^ | S2 | | |
|  |  |  |  |  | Priming (P) | | |
| 10.90^a^ | 247.02^a^ | 3.83^a^ | 19.94^a^ | 5.12^a^ | PM100 | | |
| 9.83^b^ | 215.03^b^ | 2.50^b^ | 17.43^b^ | 4.29^b^ | PH | | |
| 6.64^c^ | 151.15^c^ | 1.08^c^ | 14.19^c^ | 2.95^c^ | PD | | |
|  |  | Bacterial inoculations × Salinity levels × Priming (I × S × P) | | | | | |
| 11.19^c^ | 286.18^b^ | 4.00^ab^ | 33.88^c^ | 5.25^c-e^ | PM100 | NS | NI |
| 10.13^d^ | 257.22^f^ | 3.00^bc^ | 29.46^d^ | 4.25^d-f^ | PH |  |  |
| 7.73^gh^ | 229.42^j^ | 2.00^cd^ | 28.68^d^ | 3.25^fg^ | PD |  |  |
| 9.93^d^ | 266.22^e^ | 3.00^bc^ | 8.74^j^ | 3.25^fg^ | PM100 | S1 |  |
| 8.87^e^ | 239.12^i^ | 2.00^cd^ | 6.57^k^ | 2.25^gh^ | PH |  |  |
| 6.47^i^ | 211.22^l^ | 0.25^e^ | 4.73^l^ | 1.25^hi^ | PD |  |  |
| 8.25^fg^ | 169.26^n^ | 3.00^bc^ | 3.61^m^ | 1.25^hi^ | PM100 | S2 |  |
| 7.19^h^ | 129.09^p^ | 1.00^de^ | 2.71^n^ | 1.25^hi^ | PH |  |  |
| 6.38^i^ | 128.98^p^ | 0.25^e^ | 0.30^0^ | 1.25^hi^ | PD |  |  |
| 13.81^a^ | 299.22^a^ | 5.00^a^ | 42.88^a^ | 9.00^a^ | PM100 | NS | RI |
| 12.75^b^ | 270.31^d^ | 4.00^ab^ | 38.49^b^ | 8.00^ab^ | PH |  |  |
| 10.35^d^ | 242.22^h^ | 3.00^bc^ | 37.78^b^ | 7.00^bc^ | PD |  |  |
| 12.36^b^ | 279.20^c^ | 4.00^ab^ | 17.84^e^ | 7.00^bc^ | PM100 | S1 |  |
| 11.3^c^ | 252.22^g^ | 3.00^bc^ | 15.60^f^ | 6.00^cd^ | PH |  |  |
| 8.9^e^ | 224.09^k^ | 1.00^de^ | 13.64^g^ | 5.00^d-f^ | PD |  |  |
| 9.84^d^ | 182.08^m^ | 3.00^bc^ | 12.71^h^ | 5.00^d-f^ | PM100 | S2 |  |
| 8.78^ef^ | 142.22^o^ | 2.00^cd^ | 11.78^i^ | 4.00^e-g^ | PH |  |  |
| 0 | 0 | 0 | 0 | 0 | PD |  |  |
|  |  |  |  | Analysis of variance | | | |
| *** | *** | *** | *** | *** | I | | |
| *** | *** | *** | *** | *** | S | | |
| *** | *** | *** | *** | *** | P | | |
| *** | *** | ns | *** | * | I × S | | |
| *** | *** | ns | *** | ns | I × P | | |
| *** | *** | * | *** | ns | S × P | | |
| *** | *** | * | *** | * | I × S × P | | |
| I, S and P are bacterial inoculations, salinity levels and priming treatments. NI and RI are non-inoculation and *Rhizobium* inoculated plants. NS, S1 and S2 are non-saline, 4 and 8 dSm^−1^ of salinity stress, respectively. PM100, PH and PD are melatonin priming, hydro priming and non-priming, respectively. ns, *, ** and *** indicate non-significant and significance at 5%, 1% and 0.1%, respectively. Different letters within a column indicate means that are significantly different at P < 0.05. | | | | | | | |
